# Discontinuation of pembrolizumab for advanced urothelial carcinoma without disease progression: Nationwide cohort study

**DOI:** 10.1002/cam4.5057

**Published:** 2022-07-21

**Authors:** Katsuhiro Ito, Yuki Kita, Akira Yokomizo, Jun Miki, Yuko Yoshio, Hiroaki Matsumoto, Takehiko Segawa, Takashi Karashima, Naotaka Nishiyama, Kazuto Imai, Shigetaka Suekane, Seiji Nagasawa, Shin Higashi, Hiroyuki Nishiyama, Hiroshi Kitamura, Takashi Kobayashi

**Affiliations:** ^1^ Department of Urology Kyoto University Graduate School of Medicine Kyoto Japan; ^2^ Department of Urology Harasanshin Hospital Fukuoka Japan; ^3^ Department of Urology The Jikei University Kashiwa Hospital Chiba Japan; ^4^ Department of Urology Mie University Mie Japan; ^5^ Department of Urology Yamaguchi University Yamaguchi Japan; ^6^ Department of Urology Kyoto City Hospital Kyoto Japan; ^7^ Department of Urology Kochi Medical School Kochi Japan; ^8^ Department of Urology University of Toyama Toyama Japan; ^9^ Department of Urology Kansai Electric Power Hospital Osaka Japan; ^10^ Department of Urology Kurume University School of Medicine Fukuoka Japan; ^11^ Department of Urology Hyogo College of Medicine Hyogo Japan; ^12^ Department of Urology Hirakata Kohsai Hospital Osaka Japan; ^13^ Department of Urology University of Tsukuba Ibaraki Japan

**Keywords:** carcinoma, transitional cell, duration of therapy, immune checkpoint inhibitors, immunotherapy, pembrolizumab

## Abstract

Pembrolizumab, an anti‐programmed death 1 monoclonal antibody, has revolutionized the treatment of metastatic urothelial carcinoma. However, the optimal treatment duration for treatment responders has not been established. To address this, we retrospectively assess the treatment outcomes and duration of pembrolizumab for patients whose best response was complete response (CR) or partial response (PR) in a Japanese nationwide cohort of platinum‐refractory metastatic urothelial carcinoma. Of 203 patients whose best response was CR or PR, 83 patients discontinued pembrolizumab before progression. The median pembrolizumab treatment duration was 6.9 months. The 2‐year relapse‐free survival (RFS), treatment‐free survival, and OS rates after discontinuation were 49.0%, 57.4%, and 74.5%, respectively. CR, higher hemoglobin levels, and a better Eastern Cooperative Oncology Group performance status at the time of discontinuation were associated with significantly better RFS. Pembrolizumab was re‐administered to 12 patients. Pembrolizumab re‐challenge resulted in CR, PR, stable disease, and progressive disease in six, three, two, and one patient, respectively. Propensity score‐matched landmark analysis revealed no significant OS difference between patients who continued or discontinued pembrolizumab at 6, 12, and 18 months (*p* = 0.91, 0.99, and 0.25, respectively). Our findings demonstrated that patients with objective responses had favorable survival outcomes and suggested that pembrolizumab could be discontinued safely in this population. This study should drive further efforts to optimize the treatment duration for pembrolizumab responders.

## Graphical abstract

Favorable relapse‐free survival was observed after pembrolizumab discontinuation in patients with urothelial carcinoma, especially in those with complete responses, higher hemoglobin levels, and better ECOG‐PS at the time of discontinuation. Re‐administration upon progression was effective in 90.9% of the patients, and no difference in overall survival was observed between the patients who continued or discontinued treatment after complete or partial responses.

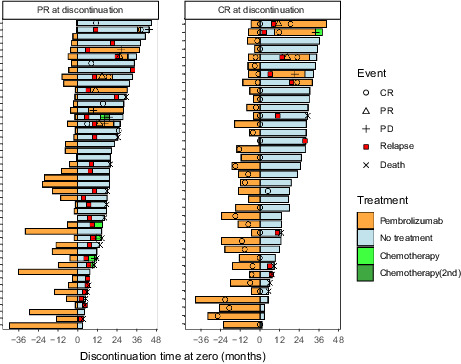

## 1 INTRODUCTION

Metastatic/unresectable urothelial carcinoma (mUC) has poor survival outcomes. Median overall survival (OS) in mUC ranges 10–12 months, and the 5‐year OS rate is less than 30%.^1^, ^2^ Recent progress in the development of immunotherapy has revolutionized the care of mUC. Pembrolizumab, an anti‐programmed death 1 (PD‐1) antibody, produced a remarkable survival benefit versus chemotherapy in the second‐line treatment of platinum‐refractory UC.^3^ Furthermore, immunotherapy can provide a long‐lasting response, the hallmark of immunotherapy, in patients with mUC. Reported clinical trial data indicated that median OS was not reached for patients with complete (CR) or partial responses (PR) after 5 years of follow‐up.^4^ These findings raise clinical questions regarding whether pembrolizumab can be safely discontinued, as well as the timing of discontinuation. In the clinical trial, patients were required to complete 2‐year pembrolizumab treatment unless the disease progressed. Meanwhile, discontinuation of treatment after a certain period for patients with a good response is common practice in clinical practice. However, data regarding drug cessation, especially in the real‐world setting, are lacking. To reduce toxicity and cost, optimal treatment duration and the criteria for treatment discontinuation should be investigated. To address this, this study aimed to clarify survival outcomes after pembrolizumab discontinuation in patients without disease progression.

## 2 MATERIALS AND METHODS

### 2.1 Patients and data collection

The clinical data were collected from the Japan Urological Oncology Group nationwide cohort of patients with platinum‐refractory mUC. The cohort characteristics and initial outcome data were reported elsewhere.^2^ Briefly, this cohort includes 755 patients who initiated pembrolizumab from August 2015 to December 2019 at 59 participating institutions. Patients who received pembrolizumab in the neoadjuvant/adjuvant setting were excluded. The person in charge of each facility evaluated responses using Response Evaluation Criteria in Solid Tumors (RECIST) version 1.1. First, we extracted the patients whose best objective response (BOR) was PR or CR. Among them, patients who discontinued pembrolizumab before disease progression were included. As these are real‐world data, the discontinuation of pembrolizumab was at the discretion of each physician and the wishes of the patient. Considering the purpose of this study, one patient who achieved CR following the resection of all metastatic lesions during pembrolizumab therapy was excluded. Patients without follow‐up data after discontinuation (*n* = 4) and those who discontinued pembrolizumab because of death from other causes (*n* = 4) were also excluded. Because the survival data were collected for the initial cohort at the end of 2019, we assessed the survival outcomes of all censored patients up to December 2021.

The study was approved by the institutional review board at Kyoto University (R1783, approved in June 2018) and review board of all institutions. This study conformed to the Declaration of Helsinki.

The primary outcome was relapse‐free survival (RFS), and the secondary outcomes were treatment‐free survival (TFS) and OS. RFS was defined as the time from treatment discontinuation to cancer progression based on RECIST, pembrolizumab retreatment, subsequent chemotherapy, or death from any cause. TFS was defined as the time from pembrolizumab discontinuation to subsequent treatment or death from any cause. The parameters evaluated in this study included age, sex, primary cancer site, histological subtype, prior surgery, and the interval since prior chemotherapy. The Eastern Cooperative Oncology Group performance status (ECOG‐PS), hemoglobin level at pembrolizumab initiation, and site of metastasis were collected at pembrolizumab initiation; after 6, 12, and 18 months of treatment; and at the time of treatment cessation.

### 2.2 Statistical analysis

All statistical analyses were performed using R (The R Foundation for Statistical Computing, version 4.0.5). Fisher's exact test and the Mann–Whitney U test were used to analyze categorical and continuous variables, respectively. The Kaplan–Meier method with the log‐rank test was used to assess RFS, TFS, and OS. We adopted landmark analysis to address immortal bias (lead‐time bias).^5^ For landmark analysis, only patients with PR or CR who were alive at each time point were included. Patients were stratified into two groups according to their drug exposure at each time point. To minimize the effect of immortal bias, patients who discontinued pembrolizumab after the time point were assigned to the control group. For propensity score matching, the propensity score was calculated by logistic regression based on age, sex, prior chemotherapy within <90 days, hemoglobin levels, ECOG‐PS, the presence of visceral and liver metastasis, and BOR at each time point. Nearest neighbor matching was performed in a one‐to‐one manner using a caliper of 0.2 of the standard deviation of the propensity score and without replacement. For time‐dependent Cox proportional hazard analysis, the timing of pembrolizumab discontinuation before progression; hemoglobin level; ECOG‐PS; presence of liver metastasis; and BOR at 0, 6, 12, and 18 months were considered time‐dependent covariates.^6^ All tests were two‐sided, and *p* < 0.05 indicated statistical significance.

## 3 RESULTS

### 3.1 Patients

**FIGURE 1:**
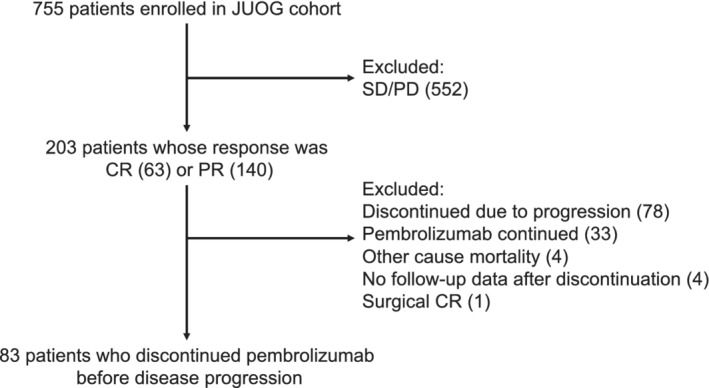
Flowchart of the patients included in this study.

Among the 203 patients whose initial response was PR or CR, 83 (40.9%) patients discontinued pembrolizumab treatment before disease progression (Figure 1). The baseline characteristics of these patients are presented in Table 1. The median pembrolizumab treatment duration was 6.9 months (interquartile range [IQR] = 4.1–14.1) and the median follow‐up duration after pembrolizumab discontinuation was 31.9 months (IQR = 24.5–40.3). The BOR at discontinuation was CR in 37 patients (44.6%) and PR in 46 patients (55.4%). The proportion of patients with primary in the upper tract was 44.6%, which is in line with Japanese subgroup in KEYNOTE‐045 trial.^7^ The primary reason for discontinuation of pembrolizumab was adverse events in 39 patients (47.0%), durable response in 39 (47.0%), and comorbidities in five (6.0%).

**TABLE 1:** Baseline characteristics of patients at pembrolizumab initiation

Patient characteristics	*N* (%) or median (interquartile range)
Age, year	73.9 (69.4–78.4)
Sex	
Male	65 (78.3)
Female	18 (21.7)
Primary site	
Ureter or renal pelvis	37 (44.6)
Bladder	46 (55.4)
Histology	
Pure UC	69 (83.1)
Mixed	14 (16.9)
Prior cystectomy or nephroureterectomy, yes	54 (65.1)
Treatment setting	
2nd line	63 (75.9)
≥3rd line	20 (24.1)
< 90 days after prior chemotherapy	37 (44.6)
Metastasis site	
Primary or lymph node only	41 (49.4)
Visceral metastasis	
Bone	10 (12.0)
Brain	1 (1.2)
Liver	7 (8.4)
Lung	24 (28.9)
Peritoneum	8 (9.6)
Hemoglobin, < 10 g/dl	26 (31.3)
ECOG‐PS	
0	53 (63.9)
1	26 (31.3)
≥2	4 (4.8)
Number of Bellmunt's risk factors (liver metastasis, hemoglobin <10 g/dl, ECOG‐PS ≥1)	
0	35 (42.2)
1	34 (41.0)
≥2	14 (16.9)

Abbreviations: ECOG‐PS, Eastern Cooperative Oncology Group performance status; UC, urothelial carcinoma.

### 3.2 Survival

**FIGURE 2:**
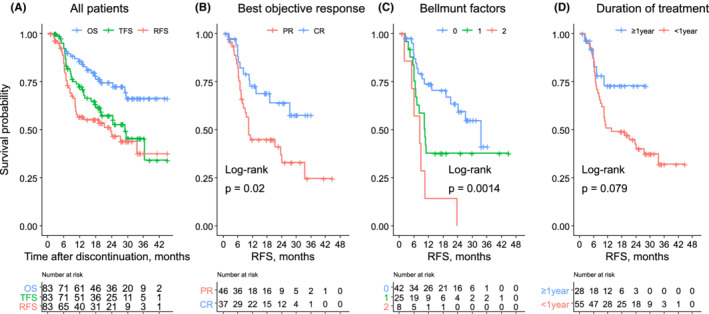
(A) Overall survival (OS), treatment‐free survival (TFS), and relapse‐free survival (RFS) of patients who discontinued pembrolizumab. RFS stratified by (B) the best objective response at the time of discontinuation, (C) number of Bellmunt factors (Eastern Cooperative Oncology Group performance status ≥1, hemoglobin <10 g/dl, and presence of liver metastasis) at discontinuation, and (D) duration of pembrolizumab treatment. PR, partial response; CR, complete response.

RFS, TFS, and OS after the discontinuation of pembrolizumab are presented in Figure 2A. Median RFS, TFS, and OS were 2.0 years, 2.4 years, and not reached, respectively. The estimated 2‐year RFS, TFS, and OS rates were 49.0% (95% confidence interval [CI] = 38.3–62.6), 57.4% (95% CI = 46.7–70.7), and 74.5% (95% CI = 64.8–85.6), respectively. Figure 2B illustrates RFS stratified by the BOR. RFS was significantly longer in patients with CR than in those with PR at the time of discontinuation (*p* = 0.02). The 2‐year RFS rates were 63.9% (95% CI = 48.5–84.3) and 37.0% (95% CI = 24.2–56.6) in patients with CR and PR, respectively. The Bellmunt risk factors (hemoglobin level < 10 g/dl, ECOG‐PS ≥1, and liver metastasis) at the start of pembrolizumab were not associated with RFS (Figure S1). Then, we investigated whether these variables at pembrolizumab discontinuation can be a prognostic factor. Intriguingly, ECOG‐PS (*p* < 0.01) and hemoglobin levels (*p* = 0.04), and the number of Bellmunt risk factors (*p* < 0.01) at pembrolizumab discontinuation were significantly associated with RFS (Figure S2; Figure 2C), which indicate the patient condition at treatment discontinuation was more important than the initial disease stage and risk factors. RFS was considerably longer in patients who received pembrolizumab for more than 1 year, although the difference was not statistically significant (Figure 2D).

**FIGURE 3:**
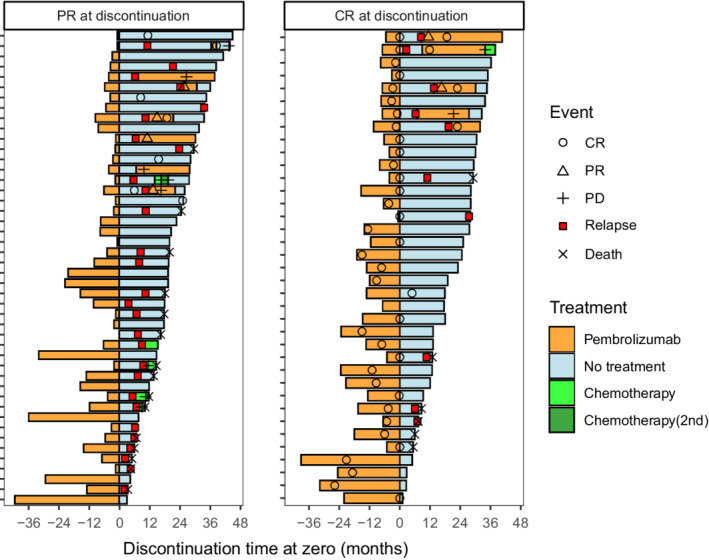
Swimmer plot after pembrolizumab discontinuation stratified by response indicating the treatment‐free duration, sequential treatments and the response, and overall survival. PR, partial response; CR, complete response; PD, progressive disease.

The clinical course after treatment cessation stratified by the BOR at the time of discontinuation is presented in Figure 3. The tumors in five patients with PR continued to shrink after pembrolizumab cessation, and they completely resolved more than 6 months later. Overall, 43 (51.8%) patients exhibited disease control, whereas 40 (48.2%) patients experienced disease relapse or death from other causes. Pembrolizumab was re‐administered to 12 patients. Pembrolizumab re‐challenge resulted in CR, PR, stable disease (SD), and progressive disease in six, three, two, and one patient, respectively. Of note, four of the five patients whose BOR for the first pembrolizumab course was CR achieved CR again. Five patients received subsequent chemotherapy, and only one had SD.

### 3.3 Comparison to treatment continuation

**FIGURE 4:**
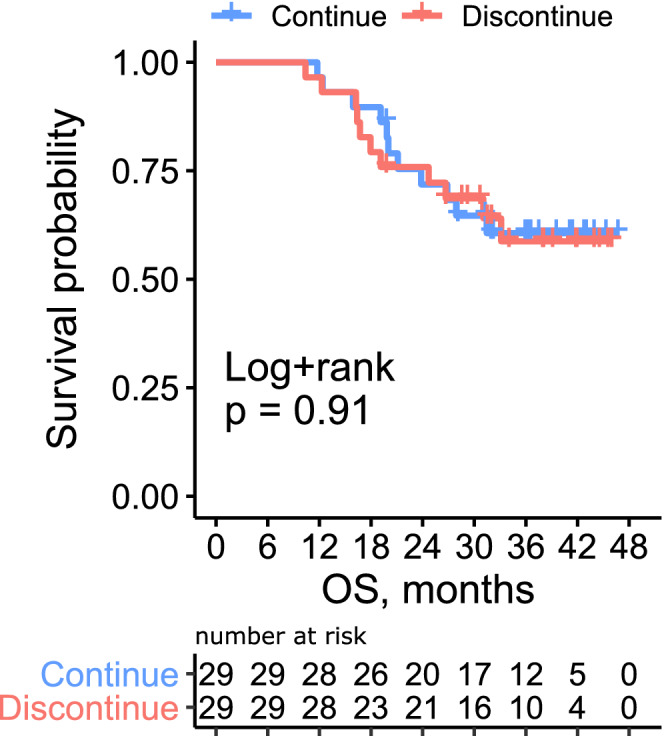
Propensity score‐matched overall survival between patients who continued or discontinued pembrolizumab at 6 months.

Because our data suggested that the patient condition at treatment discontinuation, not the initial stage, have significant effect on outcomes, we conducted propensity score‐matched landmark analysis at different time points. Patients who discontinued pembrolizumab after <6, 6–12, and 12–18 months were matched with those who continued pembrolizumab at 6, 12, and 18 months, respectively. The baseline covariates were well‐balanced between the two groups (Table S1). The OS curve is illustrated in Figure 4 and Figure S3. There was no significant difference in survival between the two groups regardless of the timing of discontinuation (*p* = 0.91, 0.99, and 0.25, respectively). To further validate this finding, we conducted time‐dependent Cox proportional hazard regression analysis (Table S2). The discontinuation of pembrolizumab before disease progression was not associated with OS (hazard ratio [HR] = 0.61, *p* = 0.07). The hemoglobin level (HR = 0.81, *p* < 0.001), ECOG‐PS (HR 1.35, *p* = 0.02), achievement of complete disease remission (HR = 0.32, *p* = 0.002) at each time point (0, 6, 12, and 18 months), and prior chemotherapy within <90 days (HR 1.66, *p* = 0.02) were significantly associated with OS.

## 4 DISCUSSION

To the best of our knowledge, this was the first real‐world study of the outcomes of patients with mUC who discontinued pembrolizumab before disease progression. We observed favorable disease control and survival even after discontinuation. CR, higher hemoglobin levels, and better ECOG‐PS at the time of discontinuation were the significant factors for prolonged response. Furthermore, patients responded to pembrolizumab re‐challenge even after disease relapse. Comparison to a matched drug continuation group indicated that treatment can be safely discontinued among responders to pembrolizumab. These observations provided new insights into the appropriate treatment duration of immune checkpoint inhibitor therapy in patients with mUC.

In this study, approximately 10% of all patients and 40% of responders (CR or PR) discontinued pembrolizumab before progression. There are no other real‐world reports of pembrolizumab interruption in UC, and it is difficult to assess whether these rates of treatment discontinuation were high. The KEYNOTE‐045 phase III trial of second‐line pembrolizumab in patients with mUC was designed to end treatment after 2 years.^1^ Among all 266 patients and 57 responders, 26 patients completed 2 years of treatment, and 10 patients discontinued pembrolizumab because a durable CR was achieved before 2 years. Similarly, the KEYNOTE‐052 study, which used pembrolizumab in the first‐line setting for platinum‐ineligible patients with mUC, reported that among 106 responders, 35 patients completed the 2‐year study and 35 discontinued treatment before progression.^8^ The protocol and clinical setting of the trial can explain the difference in results versus our observations. An elective treatment discontinuation before progression of 10%–30% was reported in real‐world analyses in other cancer types.^9^, ^10^, ^11^

We observed favorable survival outcomes after drug discontinuation, especially in patients with CR. Although the follow‐up data were immature, half of all patients and two‐thirds of patients with CR were free from treatment and progression for more than 2 years. A long‐lasting response is a hallmark of immunotherapy,^12^ and ongoing responses after treatment discontinuation have been reported in mUC^1^, ^8^ and other cancers.^10^, ^13^, ^14^ These studies also identified CR as a positive indicator of durable response. Thus, patients with CR can be good candidates for early discontinuation.

When considering pembrolizumab interruption, it is important that the efficacy of treatment is maintained. In our study, tumors that relapsed after discontinuation remained sensitive to pembrolizumab re‐challenge in most patients. Little is known about immunotherapy re‐challenge in patients with mUC.^15^ One study evaluated 13 patients, including three patients with mUC who stopped anti‐PD‐1 or anti‐programmed death ligand 1 (PD‐L1) therapy in the absence of disease progression.^16^ Two of three patients with mUC experienced tumor relapse, and they responded to the same checkpoint inhibitor (one PR and one SD) after treatment‐free periods of 19.5 and 6.5 months, respectively. Another study evaluated re‐challenge with durvalumab, an anti‐PD‐L1 antibody, in patients who completed 1 year of therapy per protocol without progression in phase I/II trials.^17^ Eight patients with mUC were included, and seven achieved PR or SD. Although further data are needed, checkpoint inhibitor re‐challenge appears to restore antitumor immunity in patients with mUC who discontinued treatment without progression.

Our observation has given us an opportunity to reconsider the appropriate treatment duration for immune checkpoint inhibitors. The current standard of care is continuous treatment for all patients until progression or up to 2 years based on FDA approval. However, our data indicated that a shorter duration of treatment might provide substantial benefits in some patients. Moreover, a long duration of treatment might increase the risk of immune‐related adverse events (irAEs) and financial difficulties. Although whether early cessation of therapy can reduce the risk of irAEs is undetermined, serious irAEs after discontinuation of immunotherapy are rare.^18^ Because immune checkpoint inhibitors are highly expensive, the economic burden and cost‐effectiveness are also important matters for patients as well as government and private healthcare groups.^19^ Several ongoing clinical trials aim to optimize the duration of immune checkpoint inhibitor therapy for metastatic melanoma.^20^, ^21^ However, the safety of stopping immunotherapy may vary by cancer type. In a non‐small‐cell lung cancer study, a 1‐year fixed duration of treatment with the anti‐PD‐1 antibody nivolumab resulted in inferior OS compared with continuous therapy.^22^ Thus, a trial to assess the mUC‐specific safety of early pembrolizumab discontinuation should be conducted. Recently, maintenance avelumab (anti‐ PD‐L1 antibody) has been approved as a new standard of care for mUC patients who maintained the initial response to platinum‐based therapies.^23^ The optimal duration of the avelumab maintenance should be also evaluated.

This study had several limitations. First, because of the retrospective nature of this cohort, patients who discontinued pembrolizumab were carefully selected by physicians. This cohort lacks precise clinical data and information about tumor size at the time of drug cessation. For patients with PR, those who discontinued may have smaller tumors than those who continued. Moreover, some patients received multimodal evaluations such as fluorodeoxyglucose positron emission tomography to confirm CR, whereas other patients had limited access to these examinations. Consequently, the characteristics of patients at pembrolizumab discontinuation were heterogeneous. Since our data are not enough to conclude early cessation in responders is safe or not, the appropriate criteria for discontinuing pembrolizumab should be prospectively examined. Our work will support the planning of trials to determine the criteria for safely stopping treatment. Second, the 2‐year follow‐up period and cohort size were not sufficient to conclude that early discontinuation is safe in patients with mUC. Because patients in this cohort remain under observation, we will provide longer‐term follow‐up results in the future. Third, although landmark analysis with propensity score matching effectively minimized selection and immortal bias, it does not achieve complete randomization. In addition, landmark analysis eliminated some patients who did not reach the different time points. Nevertheless, these cases of early progression were not the focus of this study.

In conclusion, this nationwide cohort study revealed that the survival outcomes after pembrolizumab discontinuation before progression were favorable, especially for patients with CR, higher hemoglobin levels, and better ECOG‐PS at the time of discontinuation. Tumors that relapsed after discontinuation remained sensitive to pembrolizumab in most patients. No difference in OS was observed between patients who continued or discontinued pembrolizumab after PR or CR. This study should spur further prospective trials to optimize the treatment duration for pembrolizumab responders.

## AUTHOR CONTRIBUTIONS

Katsuhiro Ito: Conceptualization, data curation, formal analysis, writing—original draft and writing—review and editing. Yuki Kita: conceptualization, data curation, writing—reviewing and editing. Akira Yokomizo, Jun Miki, Yuko Yoshio, Hiroaki Matsumoto, Takehiko Segawa, Takashi Karashima, Naotaka Nishiyama, Kazuto Imai, Shigetaka Suekane, Seiji Nagasawa, and Shin Higashi: data curation, writing—review and editing. Hiroyuki Nishiyama: Project administration, supervision. Hiroshi Kitamura: supervision. Takashi Kobayashi: supervision, conceptualization, data curation, writing—review and editing.

## FUNDING INFORMATION

The authors declare that no funds, grants, or other support were received during the preparation of this manuscript.

## CONFLICT OF INTEREST

All authors declare there is no conflict of interest.

## ETHICS STATEMENT

The study was approved by the institutional review board at Kyoto University (R1783, approved in June 2018) and review board of all institutions. This study conformed to the Declaration of Helsinki. Informed consent was obtained from all individual participants included in the study.

## Supporting information


Figure S1

Figure S2

Figure S3

Table S1

Table S2

## Data Availability

The datasets generated during and/or analyzed during the current study are available from the corresponding author on reasonable request.

## APPENDIX A

**Study institutes:** Osaka City University, Akita University, Hirosaki University, National Cancer Center Hospital, Hamamatsu University School of Medicine, Yamagata University Faculty of Medicine, Kyoto University, University of Tsukuba, Nara Medical University, Shizuoka General Hospital, University of the Ryukyus, Iwate Medical University, Oita University, Hiroshima University, Shimane University, Kansai Medical University, Osaka University, Kagawa University, University of Yamanashi, Japanese Red Cross Wakayama Medical Center, Kyoto Prefectural University of Medicine, Kobe City Nishi‐Kobe Medical Center, Japanese Red Cross Osaka Hospital, Nagoya University, Harasanshin Hospital, Hokkaido University, Japanese Red Cross Otsu Hospital, Kagoshima University, Kyushu University, Shikoku Cancer Center, Tenri Hospital, Hakodate Goryoukaku Hospital, Kitasato University, Kyoto Katsura Hospital, National Hospital Organization Kyoto Medical Center, Kumamoto University, National Hospital Organization Himeji Medical Center, Tazuke Kofukai Medical Research Institute, Kitano Hospital, Toyooka Hospital, Hokkaido Cancer Center, University of Miyazaki, Hitachi General Hospital, The Jikei University Kashiwa Hospital, Shimada Municipal Hospital, Mie University, Yamaguchi University, Ibaraki Prefectural Central Hospital, Kyoto City Hospital, Kochi School of Medicine, Ijinkai Takeda General Hospital, University of Toyama, Otsu City Hospital, Sapporo Medical University, Kansai Electric Power Hospital, Kurume University, Hyogo College of Medicine, Hirakata Kohsai Hospital, Rakuwakai Otowa Memorial Hospital, Jikei University.
