# Supplementary material for: Discontinuation of pembrolizumab for advanced urothelial carcinoma without disease progression: Nationwide cohort study
Source: Cancer Med. 2022 Jul 21;12(3):2325–32. doi: 10.1002/cam4.5057 (PMC9939199; doi:10.1002/cam4.5057)
Supplement: Supplementary file 1 — Figure S1 [file CAM4-12-2325-s001.pdf]

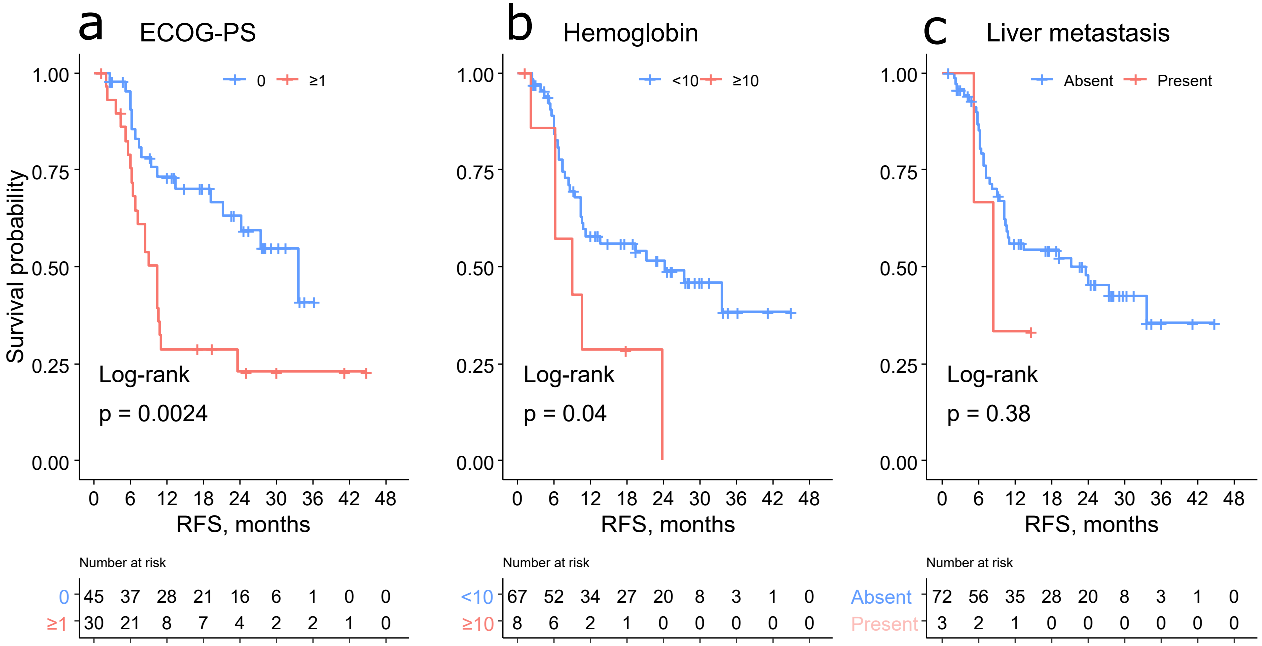

Supplementary figure 1. Relapse-free survival of patients who discontinued pembrolizumab stratified by a) ECOG-PS, b) hemoglobin, and c) presence of liver metastasis at discontinuation. ECOG-PS, Eastern Cooperative Oncology Group performance status.
