# Supplementary material for: Discontinuation of pembrolizumab for advanced urothelial carcinoma without disease progression: Nationwide cohort study
Source: Cancer Med. 2022 Jul 21;12(3):2325–32. doi: 10.1002/cam4.5057 (PMC9939199; doi:10.1002/cam4.5057)
Supplement: Supplementary file 2 — Figure S2 [file CAM4-12-2325-s002.pdf]

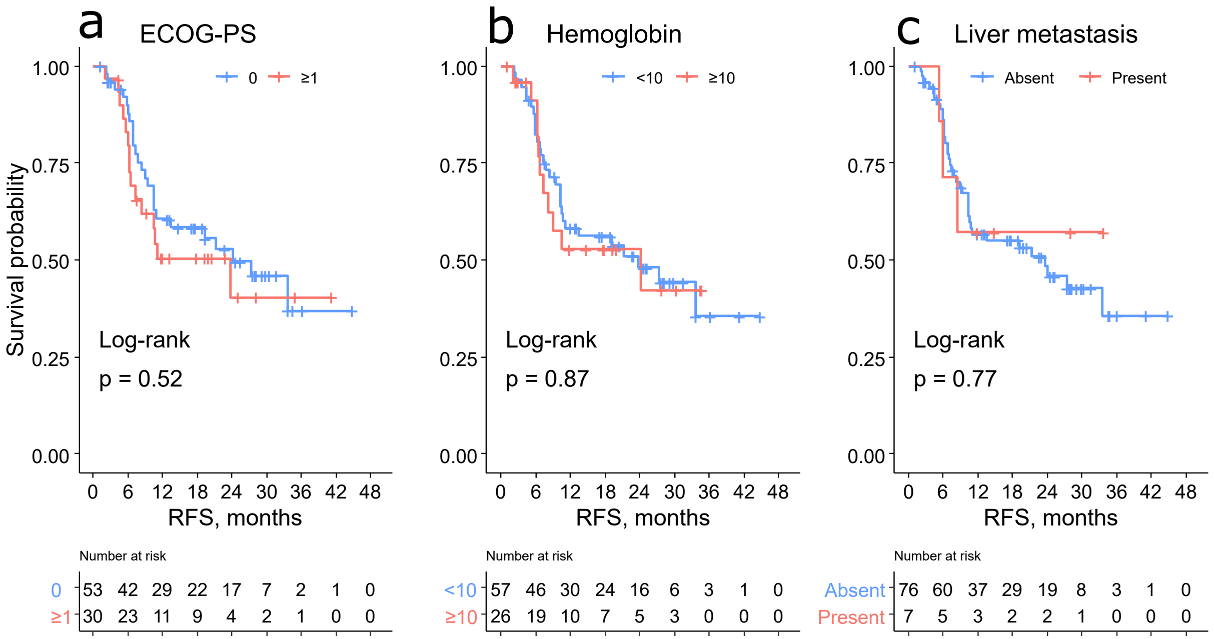

Supplementary figure 2. Relapse-free survival of patients who discontinued pembrolizumab stratified by a) ECOG-PS, b) hemoglobin, and c) presence of liver metastasis at the start of pembrolizumab. ECOG-PS, Eastern Cooperative Oncology Group performance status.
