# Supplementary material for: Discontinuation of pembrolizumab for advanced urothelial carcinoma without disease progression: Nationwide cohort study
Source: Cancer Med. 2022 Jul 21;12(3):2325–32. doi: 10.1002/cam4.5057 (PMC9939199; doi:10.1002/cam4.5057)
Supplement: Supplementary file 3 — Figure S3 [file CAM4-12-2325-s004.pdf]

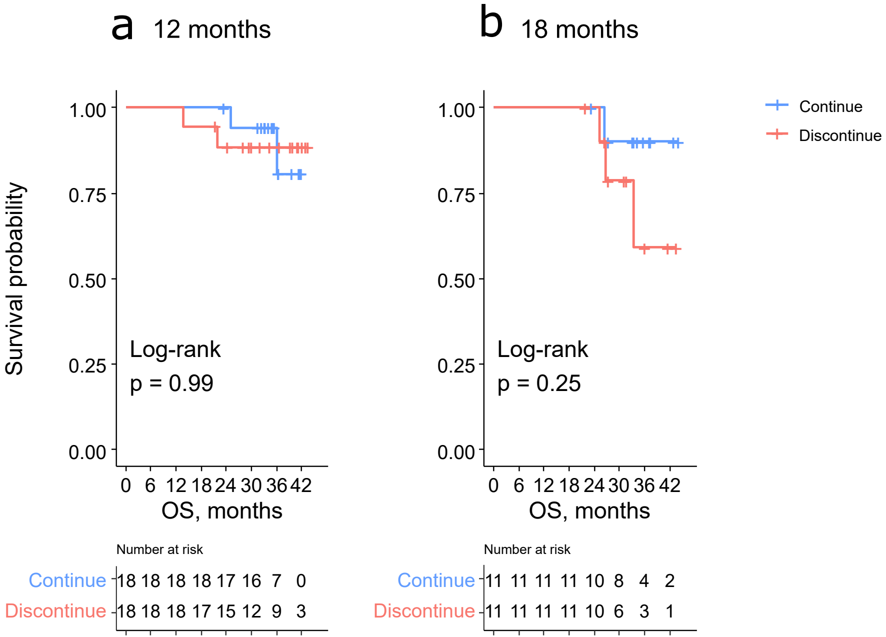

Supplementary figure 3. Propensity score-matched overall survival between patientswho continued or discontinued pembrolizumab at a) 12 months and b) 18 months.
