# Supplementary material for: Discontinuation of pembrolizumab for advanced urothelial carcinoma without disease progression: Nationwide cohort study
Source: Cancer Med. 2022 Jul 21;12(3):2325–32. doi: 10.1002/cam4.5057 (PMC9939199; doi:10.1002/cam4.5057)
Supplement: Supplementary file 4 — Table S1 [file CAM4-12-2325-s003.docx]

| **Supplementary table 1. Baseline characteristics of patients in matched cohorts** | | | | | | | | | |  |  |
| --- | --- | --- | --- | --- | --- | --- | --- | --- | --- | --- | --- |
|  | Matched at 6 months | | | Matched at 12 months | | | Matched at 18 months | | |  |  |
| Patient characteristics | Discontinued  N = 29 | Continued  N = 29 | P value | Discontinued  N = 18 | Continued  N = 18 | P value | Discontinued  N = 11 | Continued  N = 11 | P value |  |  |
| Age, year | 73.5 (70.3–76.3) | 72.7 (68.8–76.0) | 0.71 | 73.7 (68.9–76.0) | 72.5 (65.3–69.5) | 0.74 | 70.7 (67.1–74.2) | 71.2 (64.8–75.7) | 1.00 |  |  |
| Sex, male | 22 (75.9) | 20 (69.0) | 0.77 | 14 (77.8) | 15 (83.3) | 1.00 | 9 (81.8) | 8 (72.7) | 1.00 |  |  |
| < 90 days after prior chemotherapy | 15 (51.7) | 16 (55.2) | 1.00 | 4 (22.2) | 7 (38.9) | 0.47 | 4 (36.4) | 5 (45.5) | 1.00 |  |  |
| **Covariates at matched timepoint** |  |  |  |  |  |  |  |  |  |  |  |
| Visceral metastasis, yes | 8 (27.6) | 9 (31.0) | 1.00 | 3 (16.7) | 1 ( 5.6) | 0.60 | 2 (18.2) | 2 (18.2) | 1.00 |  |  |
| Liver metastasis, yes | 2 ( 6.9) | 2 ( 6.9) | 1.00 | 0 ( 0.0) | 0 ( 0.0) | 1.00 | 0 ( 0.0) | 0 ( 0.0) | 1.00 |  |  |
| Hemoglobin, g/dL | 12.7 (10.7–13.5) | 12.6 (11.3–13.2) | 0.85 | 12.5 (11.8–13.7) | 12.0 (10.9–12.9) | 0.42 | 12.4 (11.6–13.5) | 12.5 (11.7–12.9) | 1.00 |  |  |
| ECOG-PS |  |  | 0.64 |  |  | 0.47 |  |  |  |  |  |
| 0 | 19 (65.5) | 17 (58.6) |  | 11 (61.1) | 14 (77.8) |  | 9 (81.8) | 9 (81.8) | 0.14 |  |  |
| 1 | 9 (31.0) | 12 (41.4) |  | 7 (38.9) | 4 (22.2) |  | 2 (18.2) | 0 ( 0.0) |  |  |  |
| ≥2 | 1 ( 3.4) | 0 ( 0.0) |  | 0 ( 0.0) | 0 ( 0.0) |  | 0 ( 0.0) | 2 (18.2) |  |  |  |
| Best objective response, CR | 7 (24.1) | 3 (10.3) | 0.29 | 9 (50.0) | 12 (66.7) | 0.50 | 8 (72.7) | 9 ( 81.8) | 1.00 |  |  |

Data were shown as N (%) or median (interquartile range)
