# Supplementary material for: Discontinuation of pembrolizumab for advanced urothelial carcinoma without disease progression: Nationwide cohort study
Source: Cancer Med. 2022 Jul 21;12(3):2325–32. doi: 10.1002/cam4.5057 (PMC9939199; doi:10.1002/cam4.5057)
Supplement: Supplementary file 5 — Table S2 [file CAM4-12-2325-s005.docx]

| **Supplementary table 2. Cox proportional hazard model with time-dependent covariates.** | | |
| --- | --- | --- |
|  | HR (95%CI) | P value |
| Age, year | 0.99 (0.97–1.01) | 0.28 |
| Sex, male | 1.3 (0.79–2.16) | 0.31 |
| < 90 days after prior chemotherapy, yes | 1.66 (1.07–2.57) | 0.02* |
| **Time-dependent covariates** |  |  |
| Treatment discontinuation before progression, yes | 0.61 (0.35–1.05) | 0.07 |
| Liver metastasis, yes | 1.02 (0.49–2.16) | 0.95 |
| Hemoglobin, g/dL | 0.81 (0.72–0.9) | <0.001* |
| ECOG-PS | 1.35 (1.04–1.76) | 0.02* |
| Best objective response, CR | 0.32 (0.15–0.66) | 0.002* |

*　p < 0.05

CR, complete response; ECOG-PS, Eastern Cooperative Oncology Group performance status.
